# Book and Pamphlet Notices

**Published:** 1858-10

**Authors:** 


### BOOK AND PAMPHLET NOTICES.

A MANUAL OF THE PRACTICE OF MEDICINE. By T. H. TANNER, M.D., F.L.S., Author of a Manual of Clinical Medicine and Physical Diagnosis, etc., and Licentiate of the Royal College of Physicians; late Physician to the Hospital for Women, etc., etc. First American from the third revised and improved London edition. Philadelphia: Lindsay & Blakiston. 1858. Pp. 398.

The first three hundred and twenty pages of this little book is devoted to practice of medicine proper; the remainder to formula for *certain diseases.*

The terse style and brief attention to each particular disease enable the author to *go over* the most of the diseases usually treated of in a work on the practice of medicine. Dr. Tanner, in his preface, expresses the hope that his little work "may prove useful to many practitioners and students; and especially to those whose occupations prevent them from studying larger and more valuable treatises." We think that students ought not to be satisfied without studying larger and more valuable treatises, and that until they have done so—and then of course they may very properly dispense with this little work—they should neither think of, nor be allowed to practice their profession. We believe, further, that physicians whose practice has grown so large as to be unable to read larger and more valuable treatises will not need, by reason of their having more information and experience than is contained in, "this little work."

We cannot but believe that, for the good of the profession, such books ought to be discouraged. They encourage, in the lazy portion of the profession, a system of indolent routinism, discreditable to themselves and the profession at large, and extremely hazardous to the patients who trust themselves to their care. We do not wish to censure the honest labor of a brother in the profession too severely, but we must beg the privilege of speaking plainly on a matter of so much importance to the cause of correct medical education, as a treatise on the practice of medicine should be.

We have received the TRANSACTIONS OF THE NEW HAMPSHIRE

MEDICAL SOCIETY, the result of the Sixty-Eighth Anniversary meeting, held at Concord, June 1st and 2d, 1858.

As will be seen by the mention of the sixty-eighth anniversary meeting, this venerable society dates back almost to the days of the American Revolution. It is, notwithstanding its age, in vigorous manhood yet, and long may it live to do good to our noble profession. In addition to the minutes, it contains an able Valedictory Address from its retiring President, Dr. George B. Twitchell; an excellent Report of the Committee on Practical Medicine (the names of the committee are not mentioned); a Report on Surgery, consisting of some valuable cases from the pen of Dr. Dixi Crasby, by A. Smalley, M. D., of Lynn; a Paper on Carbuncle, by William Leighton, M.D., of Portsmouth; and some smaller productions by others. Dr. Twitchell thus very properly alludes to the prevailing instruments of female torture:

"Our modern fashion of dress, too, particularly with females, is a most frequent cause of disease: to what but the great weight of skirts, which the abdominal walls are obliged to sustain, are to be ascribed that so few of our women are free from uterine diseases? [Criminal abortions—ED.]

"Is it at all strange that there should be prolapsus, antiversion or retroversion in that who moves about with the direct pressure downward upon the contents of the abdomen, of from five to eight pounds of skirts?

"A few years since was gradually destroying herself, and making her life miserable by compressing the chest, and thereby destroying the functions of the lungs by tight lacing; now or quite recently she has been ruining her health and unfitting herself for carrying out the object of her creation by transferring the compression of the chest to the abdomen, thereby destroying the functions of the uterine organs. Right glad am I that instead of six or eight skirts, which heretofore has been necessary to carry out the requirements of fashion in disfiguring the human form, hoops have been introduced, for though they equally disfigure the form, they do away with the neceessity of so great weight, and therefore relieve the abdomen. Again, how many of our modern women have unfitted themselves for performing the duties of mothers by having prevented the due development of the mamma? In early childhood, before nature has thought best to give the contour of womanhood to the human breast, art steps in and by the addition of a few ounces of cotton the work is accomplished, and the girl in appearance becomes a woman! By the constant pressure of this though slight weight, the undeveloped nipple is prevented from duly forming, so that when nature has given a full supply of nourishment for the new-formed being, art has destroyed the means by which this being is to obtain the nourishment, and, as a consequence, the lactiferous ducts, not being relieved of the constantly increasing secretion, become overcharged, and inflammation, suppuration and all its attendant ills are the consequence."

The address is replete with sound sense throughout, and we should be glad to transfer more of it to our pages if space permitted.

An Essay on the Pathology and Therapeutics of Scarlet Fever. By Casper Morris, M.D., Fellow of the College of Physicians of Philadelphia, Member of the American Philosophical Society; late Lecturer on the Practice of Medicine in the Philadelphia Medical Institute, and Clinical Lecturer at the Philadelphia Hospital. Philadelphia: Lindsay & Blackiston. 1858. Pp. 173.

There is appended to this the Practical History of a New Epidemical Eruptive Miliary Fever with an Angina Ulcusculosa, which prevailed in Boston, New England, in the years 1735 and 1736; by William Douglass, M.D.

This treatise of Dr. Morris' is a well written work, gotten up for practical purposes, embodies the results of the experience and reflection of an able member of our profession, and is worthy of all respect. It should be in the hands of every practitioner who wishes to be *au fait* upon scarlet fever. Both of the above works may be had at Keene's book store on Lake street.

The Belmont Medical Journal, published at Bridgeport, Ohio, has been received and placed on our list of exchanges. It is published under the auspices of the Belmont Medical Society, for the purpose of "diffusing the proceedings of the society still more extensively, by publishing their transactions and the voluntary contributions of the members of the society." Its editors are Drs. John G. Afflick and James M. McConahey. We wish it success. W. H. B.

